# The Foreign Journals: Medical Jurisprudence

**Published:** 1838-10

**Authors:** 


### MEDICAL JURISPRUDENCE.

### *Important Cases of Death after Wounds and Injuries.*

FIRST SERIES. *Death after, but not from bodily Injuries.*

[THERE are few cases in which the value of medical evidence is more strikingly seen than those where persons die after having sustained greater or less bodily injury, and, on inspection, disease sufficient to account for death is found in one or more important organs. Who but a cautious medical practitioner is qualified to pass an opinion in such cases? and yet coroners in this country are now frequently in the habit of disposing of them without calling for any medical opinion.]

CASE I. By Dr. MEYER. A man, who had misconducted himself before a magistrate, was struck by that functionary twice on the face with the flat of his hand. One blow was slight, the other pretty severe; but neither ecchymosis nor swelling followed, and the deceased did not seem to suffer from them in any degree. He remained in the office for some minutes afterwards, signed his name to a paper, and then went home. On the next day the deceased went to his usual work, but complained of his head: his occupation was that of a miner. His companions did not observe anything particular in his manner. It was not until the thirteenth day that he sought for medical advice: he then complained much of his head, and attributed the pain which he suffered to the blows inflicted on him by the magistrate. Some medicines were prescribed for him: he passed a quiet night, but the next morning he suffered from occasional fits of the most excruciating pain. The following day, the fourteenth from the time that he was struck, he expired somewhat suddenly and unexpectedly. The magistrate was charged with having caused the death of the man by striking him on the face; and an inspection was ordered, to determine the cause of death.

The exterior of the body was free from any mark of violence. In the cranium there was no trace of fracture or of extravasation of blood; but, connected with the dura mater, on the posterior surface of the petrous portion of the right temporal bone, there was a round tumour, about the size of a hazel-nut, and of a greyish green colour. The fungous excrescence, on being opened, was found to contain a few drops of yellow coloured pus: it was connected with the bone by means of a small peduncle, but the bone was not diseased, nor was there any sign of redness or inflammation in the parts around. The tumour was evidently of long standing. The softness of the brain, from putrefaction, prevented any satisfactory inference being drawn as to its healthy or morbid condition. The thorax and abdomen presented nothing abnormal.

The inspectors, in giving an opinion as to the cause of death, observe, blows on the face may cause death, either by concussion, extravasation of blood, or by exciting inflammation of the cerebral substance: these dangerous effects are only likely to follow where the blows have been very severe; here, however, they were slight. The deceased obviously did not die of concussion, since symptoms of concussion immediately follow the violence producing it; while he remained sensible and capable of exerting himself after the violence. There was no appearance of extravasation of blood or inflammation of the brain; nor were the symptoms under which he suffered previous to death those indicative of either of these conditions. The fungus on the dura mater could not have arisen from the violence used towards the individual, fourteen days before his death: it had doubtless been forming for many months previously, and seemed to be wholly independent of any external causes. The growth of a tumour of this description is sometimes very insidious, and it often causes death suddenly by compression, when it has attained a certain size. The deceased, although he attributed the pain in his head entirely to the blows received from the magistrate, was proved to have complained at intervals of severe pain in that part for more than a year before his death. He was a man of irregular habits, and had been engaged in many quarrels, in which he had received injuries on the head. These facts rendered it highly probable that the fungus had its origin a long period prior to death. The conclusions to which the reporter of the case came were: 1, The death of the deceased did not proceed from the blows inflicted on his face by the accused. 2. Death was to be ascribed exclusively to the fungus on the dura mater, which must have been formed long antecedent to the period at which he received these blows.

On this the accused was immediately discharged from all responsibility for the death.

CASE II. By Dr. GRAFF. On the 29th November, 1835, a man, æt. 60, was struggling and fighting with another, when he was violently thrown down, and his neck compressed by his antagonist almost to strangulation. He raised himself, and wished to renew the fight, but soon afterwards fell senseless, and was obliged to be taken home. On the 2d December he felt ill, and kept his bed until the 4th. On the 6th he was up, but appeared to be wandering in his mind: he did not, however, make any complaint of illness. On the morning of the 7th he was found dead in bed: his death took place, therefore, about a week after the fight.

An inspection of the body was ordered, since it was supposed he had been killed by his antagonist. Externally there were no marks of violence, but simply a few scattered patches of lividity. In the head there was great fulness of the vessels of the brain, with general vascularity of that organ. The cavity of the chest was found to contain upwards of a pint of watery serum. The lungs were strongly adherent to the sides: they were of a dark reddish colour, partly hepatized, and partly in a state of suppuration. The pulmonary artery was ossified. In the right ventricle of the heart there was a polypus of considerable length, extending through the right auricle into the superior cava; it was fixed in the ventricle by a base of more than an inch in extent, and divided at the opposite end into four parts; it was of a firm and compact texture. A similar polypus was found in the left ventricle. The viscera of the abdomen presented no abnormal appearances. There were inguinal herniæ on both sides, and the sac on the left side contained a portion of small intestine. The medical examiners gave the following opinion in this case: 1. That serious and extensive disease, evidently of old standing, existed in some of the most important organs of the body: this referred more especially to the morbid changes in the lungs, and the polypi in the heart. 2. The deceased could not have lived long, even had he not been engaged in the fight a week before his death. 3. Had the deceased been perfectly well, the fight would probably have had no effect on him: there was no mark of external violence, except a slight sugillation on the skin of the occiput. The only conceivable danger to which he was exposed during the fight was from the compression of his neck, while lying on the ground: this might have operated by impeding the cerebral circulation. 4. The deceased died of apoplexy, and, although morbid causes existed in his body to favour the occurrence of this disease, yet, from the symptoms under which he laboured at the time of the quarrel, and subsequently, there was reason to think the fatal attack had been brought on, or at least accelerated, by the violence with which he was treated. The reporter then enters into a discussion, according to the singular principles of German jurisprudence, respecting the precise grade of mortality which should be assigned to the injuries received by the deceased. He comes to the conclusion that they must either have been *individually* mortal, (i. e. from the diseased state of the individual,) or *accidentally* mortal; but which of the two was the more likely he could not say.

[The cause of death is not here very clearly made out. The disease of the lungs was certainly sufficient to account for death; but the description of the symptoms under which the deceased laboured subsequently to the fight is so imperfect as to lead only to a conjectural opinion. The "polypi" in the heart were probably nothing more than consolidated fibrin, and were entirely of post-mortem origin. Although it may be doubtful as to what was the immediate cause of death, it seems pretty certain, from the deceased never having effectually recovered after the fight, that it was at least accelerated by the personal injuries which he had sustained.]

Case iii. By Dr. Hohnbaum. On the 6th January, 1835, the deceased, æt. 20, was violently beaten by some companions on the head and chest. A slight hemorrhage resulted from these injuries. On the same day he was taken ill, and was obliged to keep his bed. Nothing seems to have been known of his condition until the 8th April following, when he first applied for medical advice: he was then evidently labouring under diseased lungs. On the 16th May, the disease had so far advanced that all hope of his recovery was abandoned; but he lingered on until the 7th November following, when he died: *ten months* after the receipt of the injuries which, it was alleged, had caused his death. A judicial inspection of the body was ordered to be made.

Externally, there was merely cadaverous lividity. The right side of the chest appeared flattened, as it is often seen in phthisical subjects. On laying open the cavity, the right lung was found much collapsed, and placed posteriorly: it was filled with tubercles, in various states of softening and suppuration. The left lung presented traces of inflammation and strong adhesions: in its upper lobe was an abscess, containing much matter. On this side of the chest about five ounces of water were discovered. No particular appearances were met with in the head or abdomen. The result of the inspection left no doubt that the deceased had died of phthisis: but it appeared that the deceased had been in good health up to the time of the maltreatment, and it was therefore a question how far this fatal disease might have originated from the violence which it was proved he had sustained ten months previously. Against this view of the origin of the disease it was alleged that no mechanical injury had been done to the chest, sufficiently to account for the production of phthisis: there had been no fracture, laceration, or extravasation, and the inflammation found in the left lung on inspection might have been a simple accompaniment of tubercular disease. The morbid appearances indicated phthisis of long standing, and the deceased was undoubtedly of a phthisical diathesis. This disease, besides, might have been going on long before the violence, and yet have remained unperceived by his friends. The only way in which the maltreatment could have brought about the disease was by inducing chronic inflammation of the lungs in a body already predisposed to phthisis; but it is doubtful whether the violence actually operated in this manner. In conclusion, death was ascribed to phthisis proceeding from constitutional causes, the fatal effects of which had probably been accelerated by the ill treatment, as well as by the want of proper medical assistance for a long period afterwards.

[There is no doubt that in this case death was due to phthisis, and not to the violence used: the same amount of violence, applied to a non-phthisical subject, would probably have been attended with no serious effects. We cannot look upon phthisis as even likely to be a secondary consequence of mechanical injury to the chest, unless there existed a strong tendency to the disease. The appearance of phthisis under these circumstances ought to be regarded more as a coincidence than as an effect. The searching for these latent causes of death in persons who have died after having been maltreated, is a subject of great importance in legal medicine; since the prejudices of the vulgar always lead them to connect death with the bodily injuries alone, more especially where, as in this case, the disease had not strikingly manifested itself by symptoms previous to the receipt of the violence.]

Second Series. *Death from external Injury, without external Signs.*

[The following cases illustrate a very important fact in medical jurisprudence,—namely, that severe internal injuries, proving rapidly mortal, may result from mechanical violence applied to the body, without being indicated by corresponding external marks.]

Case i. By Dr. Meyer. Two men were in the act of lowering a heavy log of wood to the ground, when it slipped from their shoulders, and fell on a little girl who was walking behind them unperceived. The child was knocked down, and died in a few minutes without uttering a cry. The child was about two years and a half old. On an inspection of the body, there was not *the slightest trace of violence to be found externally.* The head was observed to be preternaturally moveable; a circumstance which was afterwards discovered to depend upon an injury to the neck. On cutting into the neck, no extravasated blood was found, but the connexion between the fifth and sixth cervical vertebræ was destroyed. The fifth vertebra had been carried forwards, and its connecting ligaments were stretched without being lacerated: there was no fracture. The spinal cord was not torn, but it had evidently been stretched; and on it, at this part, was found a coagulum of blood. No particular appearances were met with in the chest. When the abdomen was opened, about six ounces of black liquid blood escaped. On removing this, a rent, two and a half inches in length, was found in the anterior wall of the stomach, extending nearly transversely from the cardia to its fundus. The contents of the organ had become partially extravasated in the abdomen. The liver, which was large for a child, was transversely lacerated from before to behind, through the thickest part of the right lobe. No other appearances requiring notice presented themselves.

Case ii. By Dr. Hohnbaum. A child two years of age was accidentally run over by a waggon and killed. A judicial inspection of the body was ordered to be made. There were slight lacerations on the skin of the scalp, but the skin covering the chest and abdomen was entire, and that of the chest presented only a faint linear ecchymosis. The brain was normal, but its vessels and sinuses were somewhat congested. On opening the chest, the left lung was found entirely torn through in its upper lobe, the upper third of the organ hanging to the lower two-thirds only by a few fibres. The edges of the fissure were as even as if the lung had been cut with a knife: all the vessels were torn through, and about three ounces of dark liquid blood had become extravasated in the cavity. There was no fracture in any of the ribs. The heart and the viscera of the abdomen were healthy. On the left side of the chest, corresponding to the situation of the laceration through the lung, was a slight ecchymosis. The skin covering the intercostal spaces from the third to the ninth rib was of a bluish colour. With this exception the skin over the ribs and dorsal vertebræ offered not the slightest mark of external violence. There was no doubt of the injury having caused death; but the most remarkable part of the case was, the slight marks externally and the absence of fracture in the ribs coupled with so complete a laceration of the lungs. The reasons why the ribs did not break under the violence might be the following. The waggon passed quickly over the chest—perhaps in a moment. The ribs in a young child are soft, yielding, and not easily broken, on which account they may, in resisting fracture, have become pressed more closely against the lung lying beneath. The tendency to rupture in the lungs might have been increased by the terror under which the deceased laboured, having caused them to become filled with air and blood. After discussing the question as to the grade of mortality which this wound should occupy, the reporter states that it must be reckoned among the *absolutely* mortal.

[These cases are deserving of attention, especially the first. Had they occurred in England, it is pretty certain that the valuable inference to which they lead would have been wholly lost; since coroners are not in the habit of summoning medical witnesses, in cases where death is so obviously due to accident. Nevertheless, the question as to how far serious internal injuries are necessarily accompanied by marks of external violence, has been more than once raised in our courts of law; and as we may conceive has sometimes received a negative, at others an affirmative answer. Now, nothing is more certain than that most severe blows may be inflicted upon cavities, especially on the abdomen, without ecchymosis or laceration of the skin resulting. The question is one of fact and not of theory, nor is it to be answered merely from *personal* experience, the occurrence of such cases being purely accidental. Trials for causing death by a rupture of the bladder have taken place, in which the witnesses denied that a blow was struck simply because there was no ecchymosis externally: but no inference could be more erroneous; since there are many cases on record of the bladder or intestines having been lacerated by violence directly applied to the abdomen, and yet the skin had sustained no change to indicate such serious injuries. Portal and Chaussier long ago endeavoured to draw the attention of the profession to this point; but their observations seem to have been unknown to, or neglected by those who have been called on judicially to express an opinion on the subject. If the question were put in a court of law: Can a crushing force produce a laceration of the stomach and liver without leaving marks of its operation externally? many would probably answer in the negative: but the valuable case recorded by Dr. Meyer, supported as it might be by numerous others, shows that such an answer would be highly incorrect and might lead to serious consequences.]

*Henke's Zeitschrift.* 1837.

